# Non-Pharmacological Option in Postoperative Pain: Pilot Study of Intraoperative Pulsed Radiofrequency

**DOI:** 10.1093/icvts/ivag080

**Published:** 2026-03-19

**Authors:** Ryosuke Kumagai, Shinsaku Kabemura, Fumitsugu Kojima, Fujita Nobuko, Toru Bando

**Affiliations:** Department of Thoracic Surgery, St. Luke’s International Hospital, Tokyo 104-8560, Japan; Department of Thoracic Surgery, St. Luke’s International Hospital, Tokyo 104-8560, Japan; Department of Thoracic Surgery, St. Luke’s International Hospital, Tokyo 104-8560, Japan; Department of Anesthesiology, St. Luke’s International Hospital, Tokyo 104-8560, Japan; Department of Thoracic Surgery, St. Luke’s International Hospital, Tokyo 104-8560, Japan

**Keywords:** postoperative pain, pulsed radiofrequency, thoracic surgery

## Abstract

**Objectives:**

Postoperative pain remains a discomfort for patients undergoing thoracic surgery despite advances in minimally invasive techniques. Pulsed radiofrequency (PRF) is a minimally invasive neuromodulation method used for chronic pain. This pilot study aimed to evaluate the efficacy of intraoperative PRF (iPRF) as an adjunct to conventional analgesia (thoracic epidural analgesia [TEA] or intercostal nerve block [INB]) in alleviating postoperative pain and analgesic use following minimally invasive thoracic surgery.

**Methods:**

A prospective pilot cohort was compared with historical controls at a single tertiary hospital in Japan. The iPRF group (*n* = 30) received PRF targeting the intercostal nerves intraoperatively in addition to standard analgesia. The control group comprised retrospective patients who received standard analgesia alone. Patients were stratified into TEA and INB subgroups according to procedure type. The primary end-point was the proportion of patients requiring additional analgesics. Secondary end-points included pain scores (numerical rating scale [NRS]), incidence of intercostal neuralgia, and side effects.

**Results:**

Intraoperative PRF significantly reduced the need for additional analgesics in TEA (26.7% vs 60.0%, *P* = .027) and INB (6.7% vs 42.1, *P* = .020) subgroups. In the TEA group, iPRF also reduced the proportion of patients reporting NRS ≥4 following drain removal (26.7% vs 60.0%, *P* = .027). The incidence of analgesic-induced side effects was significantly lower in the iPRF INB group (0% vs 28.6%, *P* = .031). No adverse events were associated with iPRF.

**Conclusions:**

Intraoperative PRF may be a safe and effective adjunctive method for postoperative pain alleviation in thoracic surgery, reducing analgesic requirements.

**Clinical registration number:**

The Institutional Review Board of St. Luke’s International Hospital approved the study (No. 24-R085) on September 9, 2024.

## GRAPHICAL ABSTRACT

**Figure:**
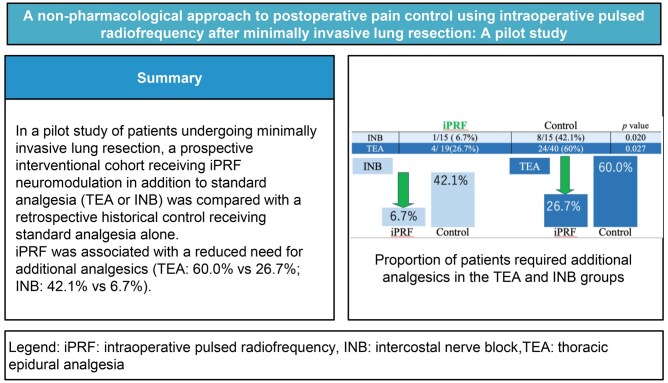

## INTRODUCTION

Postoperative pain remains a discomfort for patients undergoing thoracic surgery despite advances in minimally invasive techniques. Video-assisted thoracoscopic surgery (VATS) is the current standard treatment for lung cancer because it is associated with mild postoperative pain, short hospital stays, and faster recoveries than after traditional open thoracotomy.^1^^,^^2^ Furthermore, the implementation of fast-track surgery or enhanced recovery after surgery (ERAS) protocols in thoracic surgery has led to further improvements in postoperative outcomes.^3^^,^^4^ Despite the mild postoperative pain following VATS in comparison with thoracotomy, it is still associated with considerable pain.^5^ Post-VATS pain at the wound site and along the intercostal nerves continues to occur in 22%-63% of cases.^6^ Thoracic epidural analgesia (TEA), intercostal nerve block (INB), and paravertebral block (PVB) are widely used^7^^,^^8^; however, patients experience pain after their effects wear off shortly or when the catheter is removed. Non-steroidal anti-inflammatory drugs (NSAIDs) are commonly used to treat postoperative pain; however, they are not completely effective, and no completely effective therapy for pain relief has been established. As a result, additional analgesics are often required, and a pharmacological approach requires simultaneous management of adverse effects (eg, opioids).

**Figure 1.**
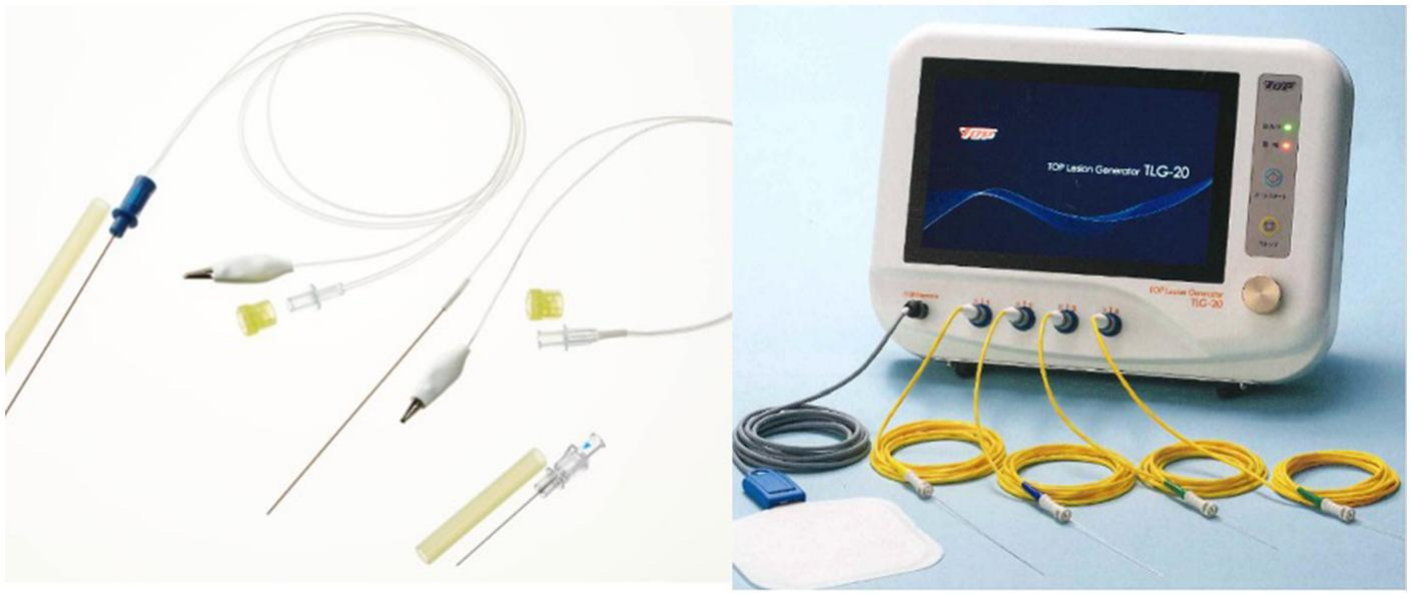
TLG-10 With Permission

Pulsed radiofrequency (PRF) is a novel therapeutic strategy that has recently been employed by pain practitioners as a non-minimally or minimally neurodestructive technique, where short bursts of high-frequency current are applied to the nervous tissue (**Figure 1**). Current is delivered in a pulse of 20 ms, followed by a silent period of 480 ms, to avoid RF heat lesions.^9^ Pulsed radiofrequency is effective in the short- or long-term pain relief of chronic pain (eg, cervical and lumbar pain and post-herpetic intercostal neuralgia), postoperative pain, and post-herpetic neuralgia.^10–15^ When applied from the tip of a needle probe placed near the target nerve, PRF provides a lasting but temporary neuromodulation effect. Only sensory nerves are affected but not motor nerves. Severe adverse effects including motor nerve paralysis are scarcely reported, and PRF use during surgery can reduce puncture pain.

The effects of PRF on acute postoperative pain remain unclear, and in this study, PRF was implemented to address the aforementioned issues on postoperative pain control. Evidence supporting the use of PRF for acute postoperative pain is limited, with only one study reported to date.^16^ This paucity of data underscores the importance of evaluating whether PRF provides additional benefits in the early postoperative period. Therefore, this study aimed to evaluate the additional pain control effect of intraoperative PRF (iPRF) when combined with conventional local analgesia (TEA or INB).

## PATIENTS AND METHODS

### Study design and population

This study was conducted as a prospective and historical control study in the pilot phase prior to a randomized controlled trial. The intervention group consisted of patients who were enrolled prospectively and underwent iPRF in addition to conventional analgesic management (iPRF group). The control group was analysed retrospectively and consisted of consecutive patients who received only conventional analgesic management between April 2023 and June 2024 (1 year prior to the start of the study) in the Department of Thoracic Surgery, St. Luke’s International Hospital, Japan.

Patients undergoing lung procedures such as partial resection, segmentectomy, or lobectomy were enrolled. Eligible patients were aged 18-85 years and scheduled for elective minimally invasive thoracic surgery, including 3- or 2-port VATS and 5-port robot-assisted thoracic surgery (RATS). Exclusion criteria were thoracotomy, emergency or urgent surgery, preoperative routine analgesic use, implanted devices affected by electrical signals, inability to take oral loxoprofen due to allergy or renal dysfunction, and inability to understand the study. Thoracotomy was excluded due to its substantially different invasiveness and clinical course. These criteria ensured uniform postoperative analgesia.

**Figure 2.**
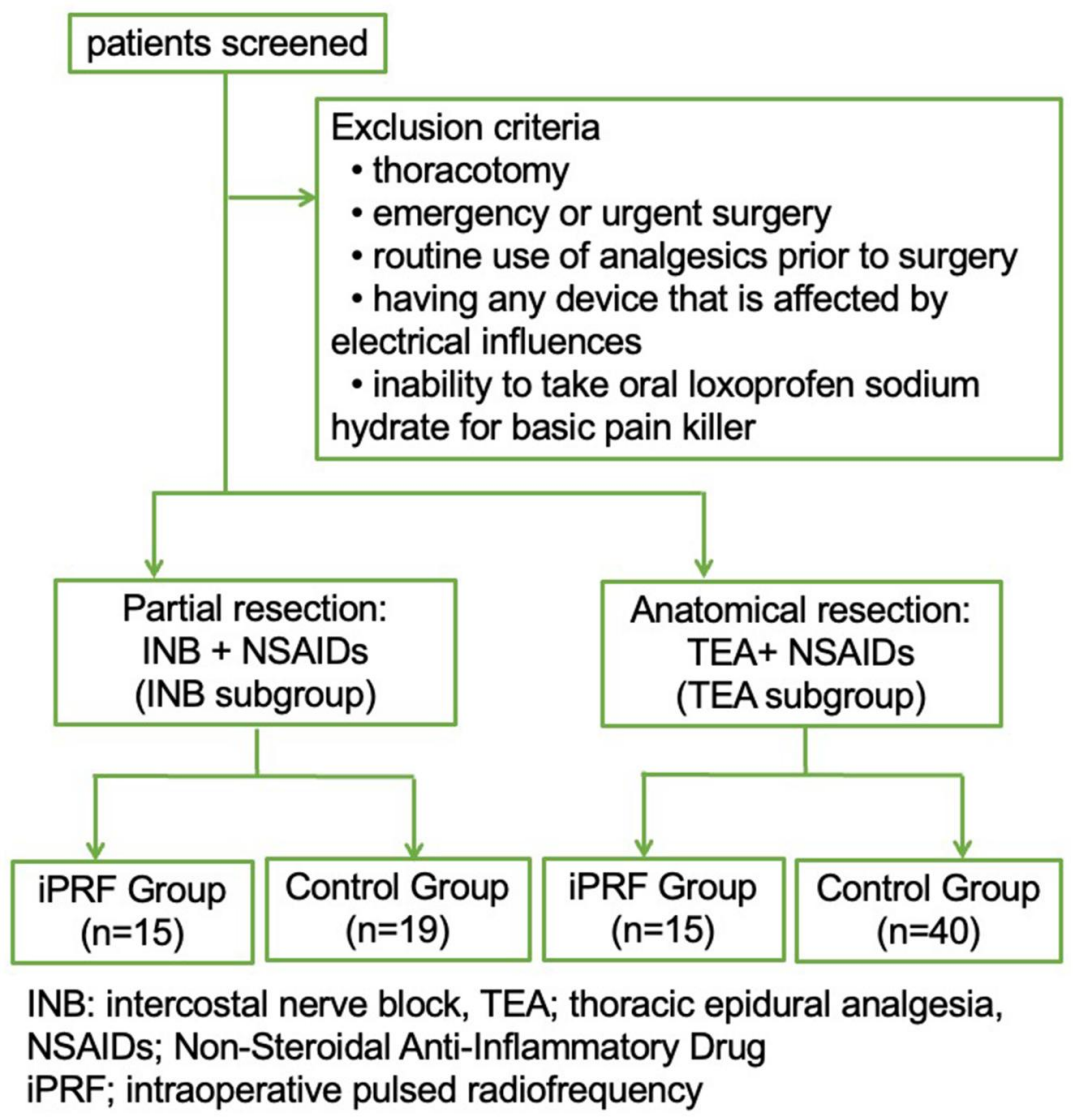
Flow Diagram. Abbreviations: INB, intercostal nerve block; iPRF, intraoperative pulsed radiofrequency; NSAIDs, non-steroidal anti-inflammatory drugs; TEA, thoracic epidural analgesia

In our department, the surgical time and intervention differ significantly between partial and anatomical resections (lobectomy or segmentectomy), so the anaesthesia methods were varied. Intercostal nerve block was used for partial resection (INB subgroup), and TEA was employed for anatomical resection (TEA subgroup). In this study, to standardize the baseline, the iPRF group and the control group were each divided into INB and TEA subgroups for comparison and evaluation (**Figure 2**).

### iPRF

**Figure 3.**
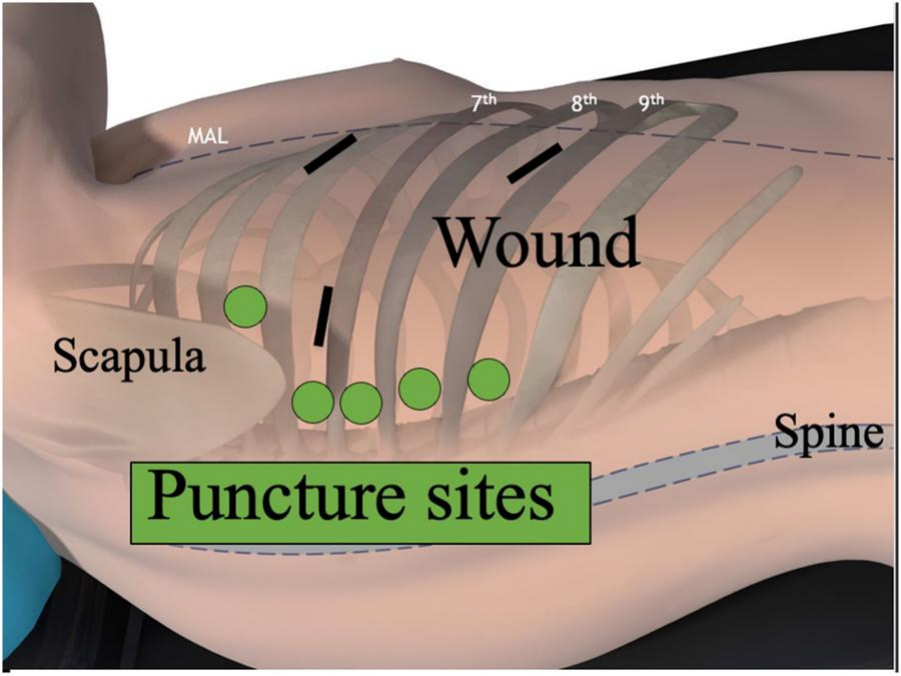
Puncture Sites for the Intraoperative Pulsed Radiofrequency in 3-Port Video-Assisted Thoracic Surgery

In the intervention group, a needle probe was inserted into the dorsal intercostal space corresponding to the surgical site, and PRF was administered intraoperatively. This technique targeted the intercostal nerves within the affected area. For example, when one wound was created in the fourth intercostal space and 2 in the seventh intercostal space, PRF was performed on the dorsal side of the wounds in the fourth to eighth intercostal muscles (**Figure 3**). Intercostal nerves were targeted using anatomical landmarks and thoracoscopic visualization, with the needle advanced as close as possible to the expected course of the nerve. The PRF settings were as follows: frequency, 2 Hz; voltage, 20 V; pulse width, 20 ms; temperature, ≤42°C; and duration, 2 min per intercostal space. Pulsed radiofrequency is performed before wound closure. Because this procedure was performed under general anaesthesia, local anaesthesia for needle probe insertion was not implemented. Pulsed radiofrequency was performed using the Top Legion Generator TLG-10 (TOP, Japan) and a dedicated needle probe (**Figure 1**).

### Surgery, anaesthesia, and postoperative care

All patients received standard care based on the ERAS protocol in the Department of Thoracic Surgery at St. Luke’s International Hospital, Japan.^17^ Experienced thoracic surgeons (2 board-certified thoracic surgeons with over 20 years of experience) performed the surgery using standardized 3- or 2- port anterior approach VATS or 5-port RATS techniques. No preoperative non-opioid analgesics were administered. In patients who underwent anatomical resection (lobectomy or segmentectomy), TEA was placed at levels 4-8 before anaesthesia induction and left in place until the day after drain removal (TEA subgroup). In patients who underwent partial resection, INB was performed before wound closure (INB subgroup). A single chest drain was placed in all patients and managed according to ERAS principles using a digital thoracic drain system (Thopaz+; Medela AG). The drain was removed in the absence of air leakage exceeding 20 mL/min for 12 consecutive hours, and the fluid discharge was non-haemorrhagic and non-chylous. Each anaesthesiologist could select the anaesthesia technique to be used during the trial. Anaesthesia was induced with fentanyl, propofol, and rocuronium and maintained with continuous infusion of propofol and remifentanil and bolus administration of rocuronium. All patients were intubated with a left-sided double-lumen tube and underwent one-lung ventilation. At the end of anaesthesia, 1 g of paracetamol was administered intravenously. The patient was extubated on the operating table. Until the start of breakfast on postoperative day 1, 1 g of paracetamol or 50 mg of flurbiprofen acetyl was administered on demand if the numerical rating scale (NRS) was ≥4 points. Early ambulation and respiratory physiotherapy were initiated soon after surgery to further support recovery within the ERAS framework. Perioperative management, nursing workflow, and discharge criteria were consistent across both study periods.

### Postoperative pain management

All patients received postoperative pain management based on a standardized clinical pathway, which included the routine use of NSAIDs. Additional analgesics were prescribed using 500 mg of paracetamol and then 50 mg of tramadol both given orally. If pain management with tramadol was inadequate, intravenous patient-controlled analgesia (IV-PCA) involving fentanyl was considered. The need for additional analgesics was determined using the NRS, a patient-reported outcome measure for assessing pain intensity. An NRS score ≥4 indicated inadequate pain management,^18^ and additional analgesics were prescribed. Even in cases with an NRS score ≥4, additional analgesics were given if the patient does not wish to receive it.

### Study end-points and assessment

The primary end-point was the proportion of patients requiring additional analgesics postoperatively during hospitalization.

The secondary end-point included the proportion of patients reporting an NRS score ≥4 during drain insertion and after drain removal; the highest NRS score on postoperative days 1, 2, 3, and 4; NRS score at discharge; NRS score at rest and during exercise during outpatient visits after discharge; number of patients who reported intercostal neuralgia during postoperative outpatient visits; and side effects. The NRS score was assessed by nurses every day in the hospital and by doctors after discharge.

### Statistical analysis

#### Sample size

The control group consisted of patients who underwent surgery in our department during the year before the study began (95 cases performed during the study period). Preliminary investigations revealed that 60% of patients from various backgrounds required additional analgesia beyond that specified in the clinical pathway. As reports on perioperative PRF are extremely limited, we referred to the only available study evaluating postoperative rescue analgesic use after PRF, in which 2 of 12 patients (16.7%) required additional medication.^16^ In our department, retrospective data from the preceding year showed that 20 of 28 eligible patients (71.4%) required additional analgesics. This contrast was used solely as a pragmatic reference to approximate a potential effect size when replacing conventional management with intraoperative iPRF. Given the limited literature and the indirect nature of this assumption, we acknowledge that this approach does not constitute a robust or definitive power calculation. Accordingly, the sample size was determined primarily based on feasibility considerations, and a target of 15 patients per group was chosen to allow preliminary estimation of the intervention effect within each clinically homogeneous cohort. This study was therefore designed as an exploratory pilot study to generate hypothesis-generating data and to inform the design of a future randomized controlled trial.

### Data analysis

For comparison between groups of continuous data, statistical significance for non-parametric data was tested using the Mann-Whitney U-test, whereas Student’s t-test was used for parametric data. *P*-values <.05 were considered significant. No missing data were encountered, and all necessary variables were retrieved from medical records. Multivariable logistic regression analyses were performed for both subgroups. The following variables were included in the multivariable model: analgesic use, age, sex, operative time, number of ports, wound size, and surgical approach (VATS vs RATS).

### Ethical statement

This prospective, historical control study was registered in the UMIN Clinical Trials Registry (R000062954). The trial began in April 2023 and ended in November 2024. The Institutional Review Board of St. Luke’s International Hospital approved the study (No. 24-R085) on September 9, 2024. All patients in the prospective iPRF intervention group provided written informed consent prior to enrolment. For the retrospective historical control group, the requirement for individual written informed consent was waived by the Institutional Review Board due to the observational and retrospective nature of the data collection. Instead, an opt-out approach was adopted, and information about the study was publicly disclosed on the institutional website in accordance with ethical guidelines. Kihachiro Arai Research Fund Research Support Program provided a research grant for this study. The investigators designed the trial and analysed the results freely, without interference from the funding source.

## RESULTS

The intervention group was enrolled in June 2024, and recruitment closed in November 2024. Among the 30 patients enrolled in the iPRF group, 15 were included in each of the TEA subgroup and INB subgroup. All patients were included for final analysis, without any dropouts. In the TEA and INB subgroups, no significant difference was found between the iPRF and control groups with respect to basic characteristics, including demographic, preoperative, and perioperative characteristics (**Table 1**).

**Table 1.** Basic Characteristics, Including Demographic and Perioperative Characteristics

	INB (partial resection)		*P*	TEA (anatomical resection)		*P*
	iPRF (*n* = 15)	Control (*n* = 19)		iPRF (*n* = 15)	Control (*n* = 40)	
Demographic characteristics						
Age, years	64.7 [63]	65.4 [61]	.94	62 [60]	65.3 [64]	.472
Male	6 (40.0%)	8 (42.1%)	.9	7 (46.7%)	15 (37.5%)	.53
Tumour size, mm	11.8 [12]	14.4 [12]	.34	21.5 [16]	21.6 [19]	.672
Perioperative characteristics						
RATS	0 (0%)	0 (0%)		4 (26.7%)	5 (15.0%)	.205
Operating time, minutes	105 [104]	91 [79.5]	.042	199 [182]	169.5 [169.5]	.285
Number of ports	2.9	2.7	.054	3.6	3.1	.106
Maximal skin incision, cm	3	2.9	.206	3.8	3.6	.251
Drainage period, days	2.13	2.12	.717	2.4	2.1	.461
Postoperative hospital stays, days	3.46	3.78	.352	4.8	4.1	.113

Values enclosed in square brackets indicate medians, while those enclosed in parentheses indicate percentages. Age and RATS are reported as absolute values, whereas all other continuous variables are expressed as means.

Abbreviations: INB, intercostal nerve block; iPRF, intraoperative pulsed radiofrequency; RATS, robot-assisted thoracic surgery; TEA, thoracic epidural analgesia.

### Use of additional analgesics

Intraoperative PRF significantly reduced the need for additional analgesics in both interventional groups compared with historical control groups without iPRF (iPRF INB, 6.7%; control INB, 42.1%; 95% CI, 0.01-0.79; *P* = .020; iPRF TEA, 26.7%; control TEA, 60.0%, 95% CI, 0.07-0.78, *P* = .027) (**Table 2**). The clinical question set as the primary end-point was validated. As a result, the use of additional analgesics for oral tramadol was greater in the control group than in the TEA group (iPRF INB, 5.3%; control INB, 6.6%: 95% CI, 0.07-22.42; *P* = .86; iPRF TEA, 0.0%; control TEA, 22.5%; 95% CI, 0.01-0.95 *P* = .044) (**Tables 3** and **4**). After multivariable adjustment, iPRF was independently associated with a reduced need for additional analgesics in both the TEA subgroup (adjusted OR 0.12, 95% CI, 0.02-0.64, *P* = .013) (**Table S1**) and the INB subgroup (adjusted OR 0.02, 95% CI, 0.0003-0.96, *P* = .048) (**Table S2**). Operative time and surgical approach were not significant in either model. After excluding RATS cases, iPRF significantly reduced additional analgesic use in the TEA group compared with controls (18.2% vs 62.9%; 95% CI = 0.03-0.67; *P* = .0097).

**Table 2.** Primary End-Point: Additional Analgesic Use (Proportion of Patients)

	iPRF (*n* = 15)	Control (*n *= 19)	95% CI	*P*
INB subgroup	1/15 (6.7%)	8/19 (42.1%)	0.01-0.79	.02
TEA subgroup	4/15 (26.7%)	24/40 (60%)	0.07-0.78	.027

Abbreviations: INB, intercostal nerve block; iPRF, intraoperative pulsed radiofrequency; TEA, thoracic epidural analgesia.

**Table 3.** Secondary End-Points: INB Group

	iPRF (*n* = 15)	Control (*n* = 19)	*P*	95% CI
NRS ≥ 4 during drain insertion	9 (60%)	14 (73.6%)	.39	0.13–2.29
NRS ≥ 4 after drain removal	3 (20%)	8 (42.1%)	.17	0.07–1.63
NRS at discharge (average)	1	0.84	.75	−0.85–1.17
Number of prescriptions of analgesics (average)	1.13	1.52	.033	−0.75–−0.03
Use of oral tramadol	1 (6.7%)	1 (5.3%)	.86	0.07–22.42
PONV	0 (0%)	5 (28.6%)	.031	0.00–1.68

Abbreviations: INB, intercostal nerve block; iPRF, intraoperative pulsed radiofrequency; NRS, numerical rating scale; PONV, postoperative nausea and vomiting.

**Table 4.** Secondary End-Points: TEA Group

	iPRF (*n* = 15)	Control (*n* = 40)	*P*	95% CI
NRS ≥ 4 during drain insertion	7 (46.7%)	11 (27.5%)	.178	0.68–7.90
NRS ≥ 4 after drain removal	4 (26.7%)	24 (60%)	.027	0.06–0.89
NRS at discharge (average)	0.6	0.925	.28	−0.95–0.30
Number of prescriptions of analgesics (average)	1.26	1.82	.015	−1.00–−0.12
Use of oral tramadol	0 (0%)	9 (2.3%)	.044	0.01–0.95
PONV	7 (46.7%)	14 (35%)	.42	0.53–5.00

Abbreviations: iPRF, intraoperative pulsed radiofrequency; NRS, numerical rating scale; PONV, postoperative nausea and vomiting.

### Post-thoracotomy pain

When comparing the pain scores over time between the iPRF and control groups, iPRF significantly reduced the proportion of patients who experienced NRS ≥4 pain after drain removal in the TEA group (iPRF INB, 20.0%; control INB, 42.1%; 95% CI, 0.07-1.63; *P* = .17; iPRF TEA, 26.7%; control TEA, 60%; 95% CI, 0.06-0.89; *P* = .027). The secondary end-points were partially validated. No significant difference was observed between the 2 groups when comparing the highest pain scores over time.

### Analgesic-induced side effects

In the INB subgroup, the number of patients who suffered from analgesics-induced side effects was significantly lower in the iPRF group (0 [0%]) than in the control group (5 [28.6%]) (95% CI, 0.00-1.68, *P* = .031) (**Table 3**). No adverse effect was observed with iPRF.

## DISCUSSION

Acute postoperative pain remains a substantial clinical challenge despite advancements in thoracic surgical techniques, such as VATS. Postoperative thoracic pain following VATS lobectomy may hinder effective coughing, leading to the retention of airway secretions, which increases the risk of pneumonia. These complications can contribute to pulmonary and cardiovascular morbidities. Moreover, inadequate pain management was found to delay recovery, being a crucial contributor to prolonged postoperative hospital stay.^19^

### Mechanism of PRF

Pulsed radiofrequency, a technology related to continuous RF, is unique in that it provides non-pharmacological pain relief without causing significant damage to the nervous tissue. The mechanism by which PRF controls pain is unclear; however, it may involve a temperature-independent pathway mediated by a rapidly changing electrical field.^20^ Pulsed radiofrequency uses RF current in short (20 ms), high-voltage bursts; the “silent” phase (480 ms) of PRF allows time for heat elimination, generally keeping the target tissue <42°C. These results not only indicate a mechanism of c-Fos activation that is independent of temperature but also hint at the inhibition of excitatory C fibres and long-term depression as a viable therapeutic mechanism in PRF.^21^

### Effectiveness of iPRF on reducing additional analgesics for postoperative pain

This study demonstrated the effectiveness of iPRF and how it compares with control in reducing additional analgesics and alleviating postoperative pain. The results of the sensitivity analyses using multivariable logistic regression were consistent with the primary findings, indicating that the observed effect of iPRF may persist even after accounting for operative and anaesthetic factors. In this study, the iPRF group required significantly fewer additional analgesics postoperatively, and in the TEA subgroup, a significantly higher proportion of patients reported NRS ≥ 4 pain during hospitalization following drain removal, suggesting that iPRF effectively alleviated postoperative pain. No significant difference was observed between the iPRF and control groups in terms of the highest NRS score daily after surgery. This is because patients who reported NRS ≥ 4 pain and requested additional analgesics were prescribed additional analgesics early on, resulting in good pain control in both groups.

In addition, the incidence of side effects induced by oral opioid analgesics was significantly lower in the iPRF group of the INB group. Although opioid use during the immediate postoperative period helps in respiratory care and physical exercise, patients who undergo lung cancer surgery are 1.3 times more likely to develop opioid addiction, particularly due to opioid overuse during the immediate postoperative period.^22^ Moreover, adjuvant treatment in addition to opioid use has even greater negative effects.^23^ Therefore, reducing postoperative opioid use is highly consequential. In the TEA subgroup, the incidence of postoperative nausea and vomiting (PONV) was higher in the iPRF group, although the difference was not significant. This may reflect improved pain control in the iPRF group while the continuous thoracic epidural fentanyl infusion was administered at the same initial rate used for patients without iPRF. Consequently, patients whose pain had already been alleviated by iPRF may have received a relatively excessive dose of epidural opioid, increasing the risk of opioid-related adverse effects such as PONV.

### Strengths and limitations

To our knowledge, this study is the first to employ iPRF following a thoracic procedure to reduce postoperative pain. This finding is considered to have high clinical importance because it provides an academic basis for significantly reducing the use of additional analgesics after VATS. In addition, the intervention is minimally invasive, which may greatly contribute to overall patient satisfaction.

The limitations of this study include the small sample size and the use of a study design comparing a prospectively enrolled intervention group with a retrospectively defined historical control group. This study was conducted as a practical preliminary pilot study prior to a randomized controlled trial. Given the early investigation stage and, correspondingly, the limited availability of eligible patients, a small sample size and simplified design were adopted to assess the feasibility of iPRF neuromodulation and to explore its potential clinical utility. The promising results obtained in this pilot study support the implementation of a larger, prospective randomized controlled trial with adequate statistical power, which is currently in the planning phase. Furthermore, the single-centre sample limits the generalizability of the findings. Moreover, participants were not blinded. This study focused primarily on short-term pain reduction; although pain relief is a very important indicator for postoperative management, long-term pain and functional indicators, such as mobility and pulmonary function, should be further analysed.

## CONCLUSIONS

Intraoperative PRF showed potential to reduce the need for additional analgesics in both intervention groups compared with historical control groups without iPRF. Intraoperative PRF may be a safe and effective adjunctive method for postoperative pain control in thoracic surgery by reducing analgesic requirements and opioid-related side effects. Intraoperative PRF showed potential to reduce the proportion of patients experiencing NRS ≥4 pain after drain removal (INB group) and to decrease adverse events. Based on these findings, iPRF may be a promising, adjunctive, non-pharmacological approach for enhancing pain control following thoracic surgery. To more clearly demonstrate the clinical efficacy of iPRF, large-scale prospective RCT trials are needed.

## Supplementary Material

ivag080_Supplementary_Data

## Data Availability

The data underlying this article will be shared on reasonable request to the corresponding author.

## ABBREVIATIONS

ERAS: enhanced recovery after surgery
INB: intercostal nerve block
iPRF: intraoperative pulsed radiofrequency
NRS: numerical rating scale
NSAIDs: non-steroidal anti-inflammatory drugs
PONV: postoperative nausea and vomiting
PRF: pulsed radiofrequency
RATS: robot-assisted thoracic surgery
TEA: thoracic epidural analgesia
VATS: video-assisted thoracoscopic surgery
