# Supplementary material for: Non-Pharmacological Option in Postoperative Pain: Pilot Study of Intraoperative Pulsed Radiofrequency
Source: Interdiscip Cardiovasc Thorac Surg. 2026 Mar 19;41(3):ivag080. doi: 10.1093/icvts/ivag080 (PMC13032819; doi:10.1093/icvts/ivag080)
Supplement: ivag080_Supplementary_Data [file ivag080_supplementary_data.docx]

Supplementary Table 1. Additional analgesic use (proportion of patients) after adjustment for the predefined covariates TEA group

| **Variable** | **Adjusted Odds Ratio** | **95% Confidence Interval** | **P value** |
| --- | --- | --- | --- |
| iPRF | 0.12 | 0.02–0.64 | 0.013 |
| Age | 0.97 | 0.91–1.02 | 0.259 |
| Male sex | 2.19 | 0.59–8.16 | 0.245 |
| Operative time | 1.01 | 0.99–1.03 | 0.144 |
| Number of ports | 0.32 | 0.04–2.28 | 0.255 |
| Wound length | 3.08 | 0.96–9.82 | 0.058 |
| RATS (vs VATS) | 2.99 | 0.21–43.4 | 0.423 |

Supplementary Table 2. Additional analgesic use (proportion of patients) after adjustment for the predefined covariates INB group

| **Variable** | **Adjusted OR** | **95% Confidence Interval** | **P value** |
| --- | --- | --- | --- |
| iPRF | 0.02 | 0.0003 – 0.96 | 0.048 |
| Age | 0.87 | 0.70 – 1.07 | 0.19 |
| Male sex | 0.004 | 0.000001 – 9.85 | 0.16 |
| Operative time | 1.06 | 0.94 – 1.19 | 0.36 |
| Number of ports | >1×10¹⁰ | 0 – ∞ | 1.00 |
| Wound length | 29.7 | 0.13 – 6849 | 0.22 |
